# Supplementary material for: Variation in species diversity and functional traits of sponge communities near human populations in Bocas del Toro, Panama
Source: PeerJ. 2015 Nov 5;3:e1385. doi: 10.7717/peerj.1385 (PMC4647605; doi:10.7717/peerj.1385)
Supplement: Table S1 [file peerj-03-1385-s001.docx]

|  |  |  |  |  |
| --- | --- | --- | --- | --- |
| Table S1: GenBank accession numbers of the gene sequences coding for the small (18S) and large (28S) nuclear ribosomal subunits used in tests of phylogenetic relatedness. | | | | |
|  |  |  |  |  |
| Sponge Species | 18S accession number | Sequence Length (bp) | 28S accession number | Sequence Length (bp) |
| *Agelas conifera* | AY734443 | 1798 | KC869634 | 3094 |
| *Aiolochroia crassa* | KC901954 | 1712 | KC869494 | 2026 |
| *Amphimedon compressa* | KC902400 | 1667 | JN178945 | 583 |
| *Aplysina cauliformis* | KC902201 | 1677 | KC869470 | 3100 |
| *Aplysina fulva* | KC902200 | 1711 | KC869518 | 3143 |
| *Aplysina lacunosa* | KC901897 | 1681 | KC869566 | 3068 |
| *Callyspongia vaginalis* | EU863813 | 417 | EU863806 | 368 |
| *Chondrilla caribensis* | KC901951 | 1723 | KC869604 | 3181 |
| *Cinachyrella kuekenthalli* | EU702414 | 1758 | KC869490 | 2318 |
| *Cliona delitrix* | NA |  | KC869510 | 3102 |
| *Desmapsama anchorata* | HE591468 | 1765 | KC952730 | 386 |
| *Ectyoplasia ferox* | EU702415 | 1794 | KC869540 | 3192 |
| *Erylus formosus* | KC902118 | 1685 | NA |  |
| *Geodia neptuni* | AY737635 | 1802 | NA |  |
| *Haliclona walentinae* | KC902131 | 1719 | JN178946 | 614 |
| *Iotrochota birotulata* | KC902147 | 1669 | AY561884 | 1519 |
| *Ircinia campana* | KC902359 | 1715 | KC869531 | 3133 |
| *Ircinia felix* | KC902297 | 1707 | NA |  |
| *Ircinia strobilina* | KC902103 | 1716 | KC869580 | 2074 |
| *Lissodendoryx colombiensis* | KC902105 | 1728 | KC869647 | 2360 |
| *Monanchora arbuscula* | KC902187 | 1718 | KC869447 | 3121 |
| *Mycale laevis* | NA |  | KC869556 | 3141 |
| *Mycale laxissima* | KC902345 | 1726 | KC961715 | 387 |
| *Neopetrosia carbonaria* | KC902003 | 1690 | KC869628 | 2373 |
| *Neopetrosia proxima* | KC902252 | 1716 | NA |  |
| *Neopetrosia rosariensis* | KC902399 | 1877 | KC869499 | 3410 |
| *Neopetrosia subtriangularis* | NA |  | KC869609 | 3394 |
| *Niphates erecta* | KC902280 | 1736 | JN178944 | 610 |
| *Plakortis angulospiculatus* | EU702423 | 1996 | NA |  |
| *Plakortis halichondrioides* | HM118543 | 1724 | KC869492 | 3091 |
| *Smenospongia aurea* | AY591806 | 1799 | NA |  |
| *Spirastrella hartmani* | NA |  | KC869504 | 3128 |
| *Spongia pertusa* | KC902365 | 1715 | KC869488 | 3131 |
| *Svenzea cristinae* | KC902041 | 1720 | NA |  |
| *Svenzea zeai* | KC902075 | 1692 | KC869635 | 3139 |
| *Verongula reiswigi* | KT943914 | 584 | NA |  |
| *Verongula rigida* | NA |  | KC869452 | 3141 |
| *Xestospongia bocatorensis* | KC902039 | 1875 | NA |  |
| *Xestospongia muta* | KC902281 | 1502 | NA |  |
