# Supplementary material for: Variation in species diversity and functional traits of sponge communities near human populations in Bocas del Toro, Panama
Source: PeerJ. 2015 Nov 5;3:e1385. doi: 10.7717/peerj.1385 (PMC4647605; doi:10.7717/peerj.1385)
Supplement: Supplemental Information 1 [file peerj-03-1385-s002.docx]

**Supplemental Methods**

*DNA extraction-PCR amplification-Sequencing.* Genomic DNA was extracted from ethanol-preserved samples of 4 individuals of *Verongula reiswigi* using the Wizard Genomic DNA Purification Kit (Promega), following the manufacturer’s protocol. The oligonucleotide primers SP18cF and SP18dR (Redmond et al. 2013) were used to amplify a portion of the 18S ribosomal subunit, yielding an approximately 650bp fragment, while the oligonucleotide primers SP635F and SP1411R were used to amplify a portion of the 28S ribosomal subunit (Thacker et al. 2013), yielding an approximately 750bp fragment. PCR reaction products were gel-purified and cleaned using the Wizard PCR Preps DNA Purification System (Promega). Forward and reverse sequencing reactions were performed at the University of Alabama at Birmingham (UAB) Center for AIDS Research (CFAR) DNA Sequencing Core Facility. Forward and reverse sequences were compared to ensure the accuracy of sequencing reactions in CodonCode Aligner software (CodonCode), yielding a final consensus sequence for each voucher specimen.

*Chlorophyll a analysis.* Chlorophyll *a* concentration was used to estimate photosymbiont abundance and as a functional trait in the current study. Most chlorophyll *a* concentrations for the surveyed species were inferred from Erwin and Thacker (2007), which assessed chlorophyll *a* concentrations of 60 of the most abundant species in the Bocas del Toro region. However, one dominant species in our surveys, *Svenzea cristinae*, was not represented in Erwin and Thacker (2007), and chlorophyll *a* concentration was measured according to the methods described in Freeman and Thacker (2011). Briefly, 0.25 g of frozen tissue was lyophilized overnight, weighed to the nearest 0.001 g, and added to a 20-mL scintillation vial covered with aluminum foil to prevent photo-degradation of chlorophyll. Samples were placed in 5 mL of 90% acetone and extracted for 18 h at 4°C. Afterwards, 1.5 mL of the extract was transferred to a quartz spectrophotometric cuvette, and absorbencies were measured at 750, 664, 647, and 630 nm. Concentrations of chlorophyll *a* were determined from these absorbencies and formulas from Parsons, Maita, & Lalli (1984) and standardized to sponge dry mass.
