# Supplementary figures and images for: Variation in species diversity and functional traits of sponge communities near human populations in Bocas del Toro, Panama
Source: PeerJ. 2015 Nov 5;3:e1385. doi: 10.7717/peerj.1385 (PMC4647605; doi:10.7717/peerj.1385)

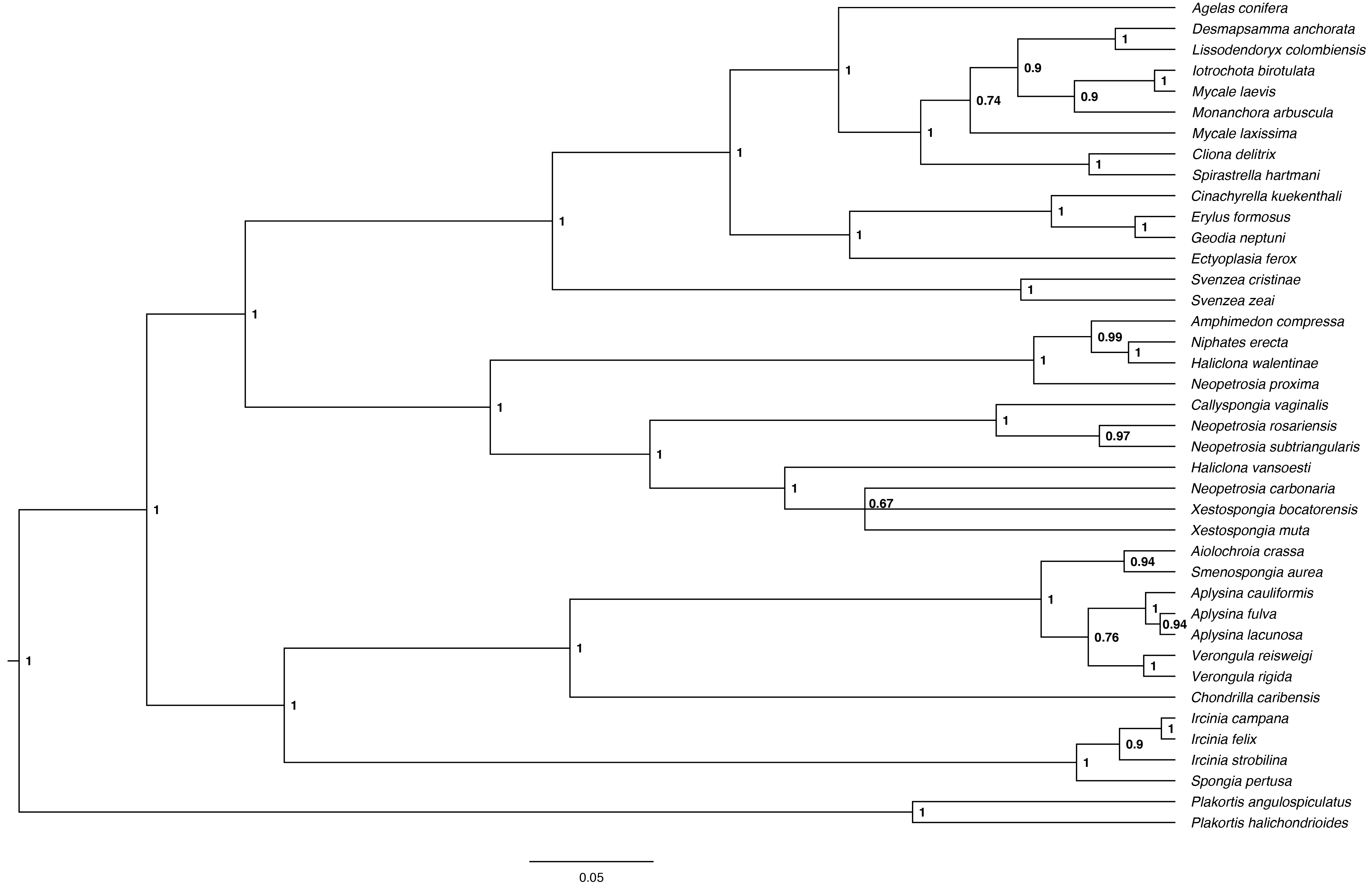

Supplement: Figure S1 — Bayesian phylogeny of sponge species observed within all transects constructed from a partitioned alignment of 18S and 28S nuclear ribosomal subunit sequences. Node values represent Bayesian posterior probability (range = 0–1) with those closer to “1” representing higher clade credibility. [file peerj-03-1385-s003.png]
